# Supplementary material for: Transcriptomic and Metabolomic Analysis Reveals the Potential Roles of Polyphenols and Flavonoids in Response to Sunburn Stress in Chinese Olive (Canarium album)
Source: Plants (Basel). 2024 Aug 25;13(17):2369. doi: 10.3390/plants13172369 (PMC11397064; doi:10.3390/plants13172369)
Supplement: Supplementary file 1 [file plants-13-02369-s001.zip › supplemental Figures.pdf]

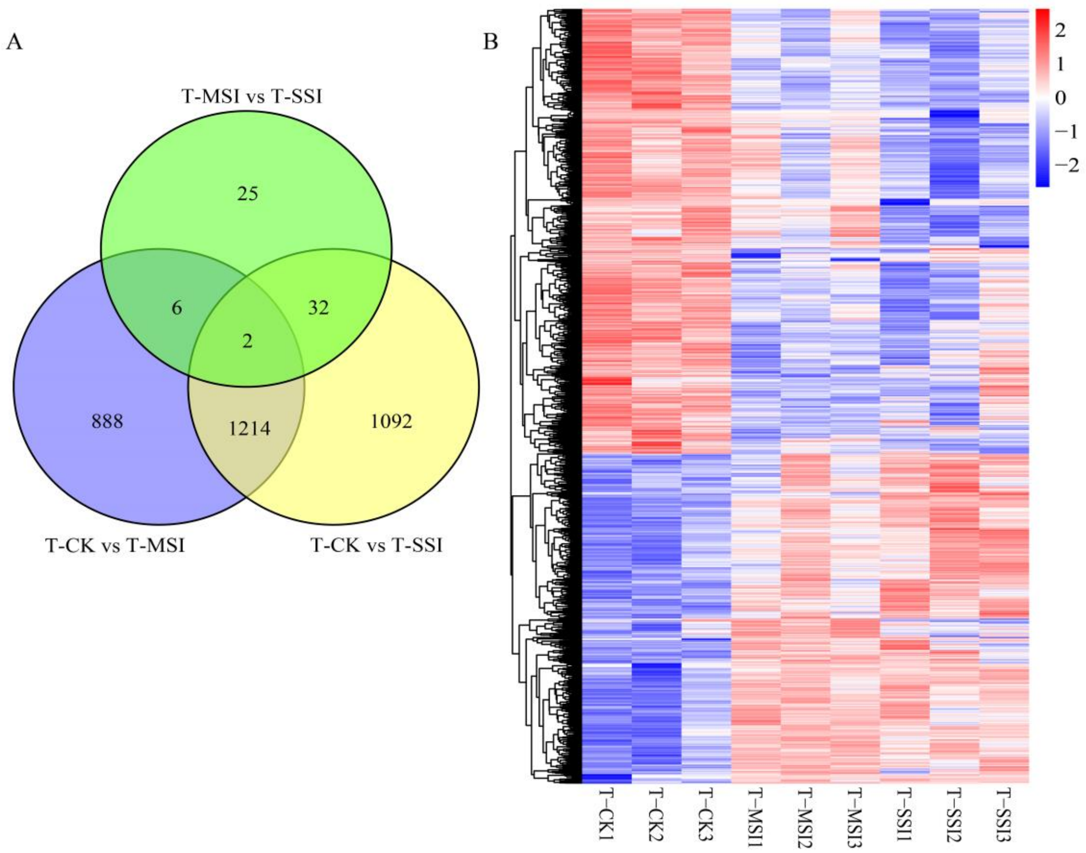

Figure S1. DEGs identification. (A) and (B) indicate the venn diagram and cluster analysis. T-CK, T-MSI, and T-SSI indicate the transcriptomes of control check, mild sunburn injury, serious sunburn injury, respectively.

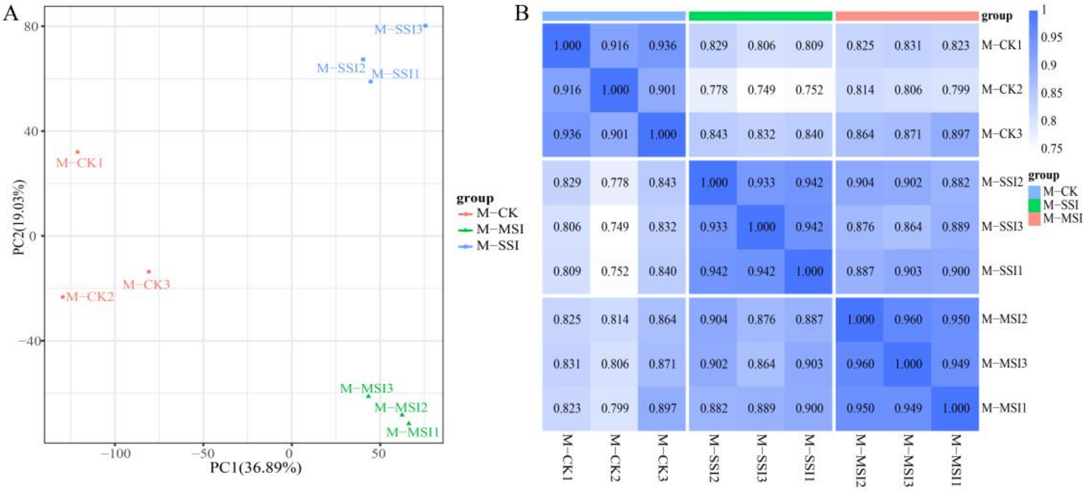

Figure S2. Metabolome data assessment of Chinese olive fruits. (A) and (B) indicate the PCA analysis and Spearman's correlation coefficient analysis. M-CK, M-MSI, and M-SSI indicate the metabolomes of control check, mild sunburn injury, serious sunburn injury, respectively.

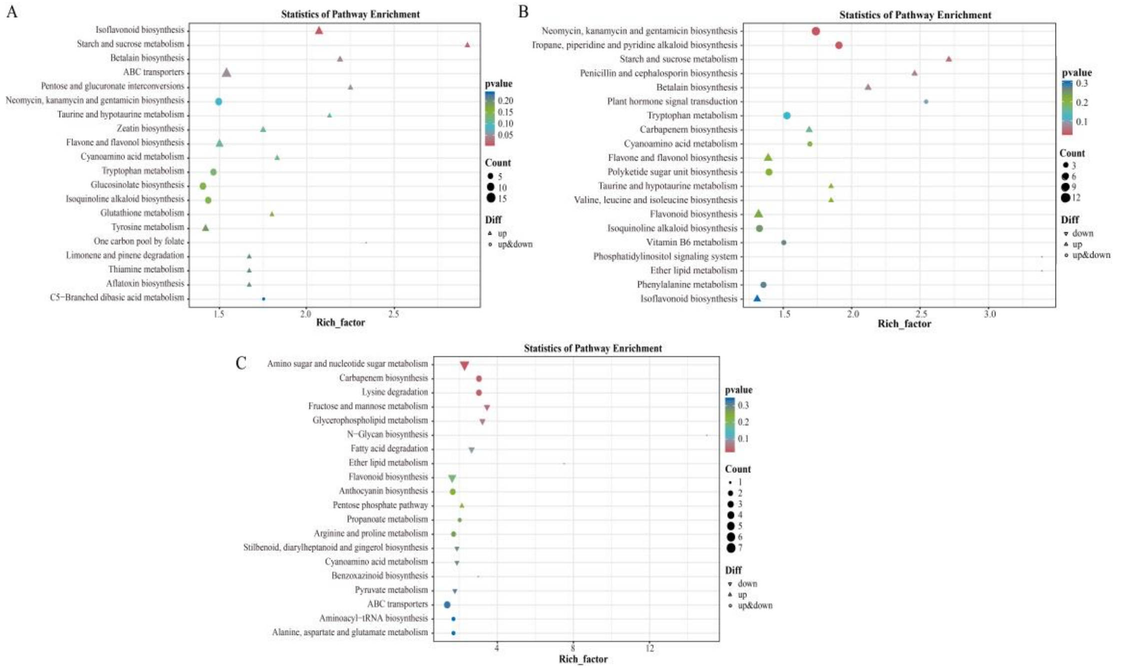

Figure S3. DAM-based enrichment analysis of KEGG pathway. (A), (B), and (C) indicate the comparisons of M-CK vs M-MSI, M-CK vs M-SSI, and M-MSI vs M-SSI.
