# Supplementary material for: Transcriptomic and Metabolomic Analysis Reveals the Potential Roles of Polyphenols and Flavonoids in Response to Sunburn Stress in Chinese Olive (Canarium album)
Source: Plants (Basel). 2024 Aug 25;13(17):2369. doi: 10.3390/plants13172369 (PMC11397064; doi:10.3390/plants13172369)
Supplement: Supplementary file 1 [file plants-13-02369-s001.zip › Table S7 The 20 days' meteorological conditions before sampling.pdf]

Table S7 The 20 days' meteorological conditions before sampling

Date	Daily precipitation/mm	Daily average temperature/℃	Daily maximum temperature/℃	Daily minimum temperature/℃	Sunshine hours/h
2022/7/10	0.0	32.6	39.1	27	11.6
2022/7/11	0.0	32.5	38.6	27.3	9.8
2022/7/12	0.0	32.4	39.9	27.3	10.4
2022/7/13	0.0	31.6	36.2	27.1	10.6
2022/7/14	0.0	31.3	36.5	26.4	6.9
2022/7/15	0.0	31.7	37.9	27.1	9.6
2022/7/16	0.0	31.6	37.8	27	7.9
2022/7/17	0.0	31.3	38.1	27.4	3.6
2022/7/18	15.8	27.9	34.2	24.6	6.2
2022/7/19	0.0	29.0	34.4	24.9	10.2
2022/7/20	0.0	31.6	39.4	26.6	12.7
2022/7/21	0.0	32.6	39.6	27	12.3
2022/7/22	0.0	32.7	40.3	27	12.5
2022/7/23	0.0	32.6	41.1	27	12.6
2022/7/24	0.0	32.9	41.3	27	8.4
2022/7/25	0.0	32.1	38.4	27.5	9.6
2022/7/26	0.1	31.6	37.7	26.9	7.3
2022/7/27	0.2	31．0	36.0	27.3	6.9
2022/7/28	0.0	31.2	37.0	27.3	9.8
2022/7/29	0.0	31.4	36.9	27.1	9.8
